# A Systematic Review of International Affective Picture System (IAPS) around the World

**DOI:** 10.3390/s23083866

**Published:** 2023-04-10

**Authors:** Diogo Branco, Óscar F. Gonçalves, Sergi Bermúdez i Badia

**Affiliations:** 1 Faculty of Exact Sciences and Engineering (FCEE) & Madeira N-LINCS, University of Madeira, Caminho da Penteada, 9020-105 Funchal, Portugal; 2 Agência Regional para o Desenvolvimento de Investigação, Tecnologia e Inovação (ARDITI), Caminho da Penteada, 9020-105 Funchal, Portugal; 3 Proaction Laboratory, CINEICC, Faculty of Psychology and Educational Sciences, Colégio de Jesus, University of Coimbra, R. Inácio Duarte 65, 3000-481 Coimbra, Portugal

**Keywords:** IAPS, review, emotion, physiological measures

## Abstract

Standardized Emotion Elicitation Databases (SEEDs) allow studying emotions in laboratory settings by replicating real-life emotions in a controlled environment. The International Affective Pictures System (IAPS), containing 1182 coloured images as stimuli, is arguably the most popular SEED. Since its introduction, multiple countries and cultures have validated this SEED, making its adoption on the study of emotion a worldwide success. For this review, 69 studies were included. Results focus on the discussion of validation processes by combining self-report and physiological data (Skin Conductance Level, Heart Rate Variability and Electroencephalography) and self-report only. Cross-age, cross-cultural and sex differences are discussed. Overall, IAPS is a robust instrument for emotion elicitation around the world.

## 1. Introduction

Emotions are complex psychological phenomena that play a key role in interacting with and perceiving the world. Over the years, researchers have offered multiple definitions of emotion. Although some slight differences are found in these definitions, the convergent point is that an emotional experience is a product of three factors: a subjective experience, physiological changes, and behavioral expressions in response to a situation [1,2]. Emotions can be represented in the emotional space. The distribution of emotions in the emotional space has two major concurrent perspectives: discrete and dimensional (continuous). The discrete perspective suggests that emotions are limited to basic categories and each emotion is distinct and separated from another. Examples of basic theories are Ekman’s six basic emotions (anger, disgust, fear, happiness, sadness, and surprise) [3] and the Plutchik (1980) model, with eight basic emotions (anger, anticipation, joy, trust, fear, surprise, sadness, and disgust) [4]. The dimensional perspective suggests that emotions are a continuum of valence (pleasant–unpleasant) and arousal (calm–excited), with each emotion being described as a point in the emotional space. An example of dimensional perspective is Russel’s (1980) circumplex model of affect, in which emotions are organized in a circular space. This circular space is divided into four quadrants, with a horizontal axis corresponding to valence and a vertical axis corresponding to arousal. The emotion location reflects the amount of valence and arousal [5].

In order to study emotions in a systematic and controlled manner, Standardized Emotion Elicitation Databases (SEEDs) are needed. These are a set of databases used for emotion elicitation that allow the replication of real-life emotion in a controlled environment. SEEDs are available in various formats, such as images [6,7,8], videos [9,10,11], audio [12,13,14], text [15,16,17], or 3D objects [18,19,20]. The validation process of these varies between a combination of self-report and physiological data (e.g., Skin Conductance Level (SCL), Heart Rate Variability (HRV) and Electroencephalography (EEG)), as well as self-report only. Self-report can be dimensional by using the Self-Assessment-Manikin (SAM), a nine-point Likert-type scale with three dimensions (valence, arousal, and dominance) and/or categorial, in which participants have to categorize the stimuli in one or multiple emotions [21].

The most used SEED is the International Affective Picture System (IAPS), comprising 1182 coloured pictures from various semantic subjects distributed along the affective space (dimensional). These images are distributed along 20 sets, each with roughly 60 images [22]. The IAPS was first introduced in 1997 and updated with more images in each iteration. In the normative studies, groups of participants (adults and children) were in a room with similar lighting conditions and rated the projected images in valence, arousal, and dominance using SAM. Furthermore, studies using physiological measurements demonstrated a congruency between the self-report and physiological data, solidifying it as a reliable tool for emotion elicitation [23,24]. In Lang’s studies, the “boomerang shape” was reported. This emerges from the resemblance with a boomerang of the distribution plot of the non-linear relationship between valence and arousal ratings. Some studies used in this review identified the presence of the boomerang shape as an indicator of the fitness of the IAPS [22].

Since its creation and original validation, IAPS has impacted multiple fields. Some studies used IAPS pictures for paradigms such as N-back, GoNoGo, and Task Switching to study working memory, inhibitory control, and cognitive flexibility [25,26,27] (N-Back is a cognitive task in which participants are presented with a sequence of stimuli (e.g., letters, numbers, or images) and are asked to indicate whether the current stimulus matches the stimulus presented in trials earlier [28]. This task is used as an assessment of working memory capacity and executive function. The Go/NoGo task is used to measure inhibitory control, in which participants are presented with a series of stimuli (e.g., letters, numbers, or images) and instructed to respond only to certain stimuli (Go trials) and withhold responses to others (NoGo trials) [29]. In the task-switching paradigm, participants are presented with a series of tasks. Commonly, participants switch between two tasks that compete for cognitive resources (e.g., color words are written with incongruent ink color, and participants are asked to name the ink color rather than the word) [30]). IAPS has also been used to study mental disorders [31,32]. In the affective computing field, IAPS is used for the model creation by exploring the relationship between computer vision (CV) features (e.g., color, texture, shape) and emotion. These models can be integrated in emotional-based image retrieval (EBIR) systems by using CV features with emotional labels (e.g., positive, negative, or neutral) for the retrieval and evaluation of new images [33].

Finally, with this review, we aim to provide an overview of the state of the art of IAPS validations by comparing the studies in terms of sample size, picture selection criteria, and measurement techniques. Furthermore, we aim to provide additional recommendations for using the IAPS in future research, considering patterns or discrepancies across studies.

## 2. Materials and Methods

**Figure 1:**
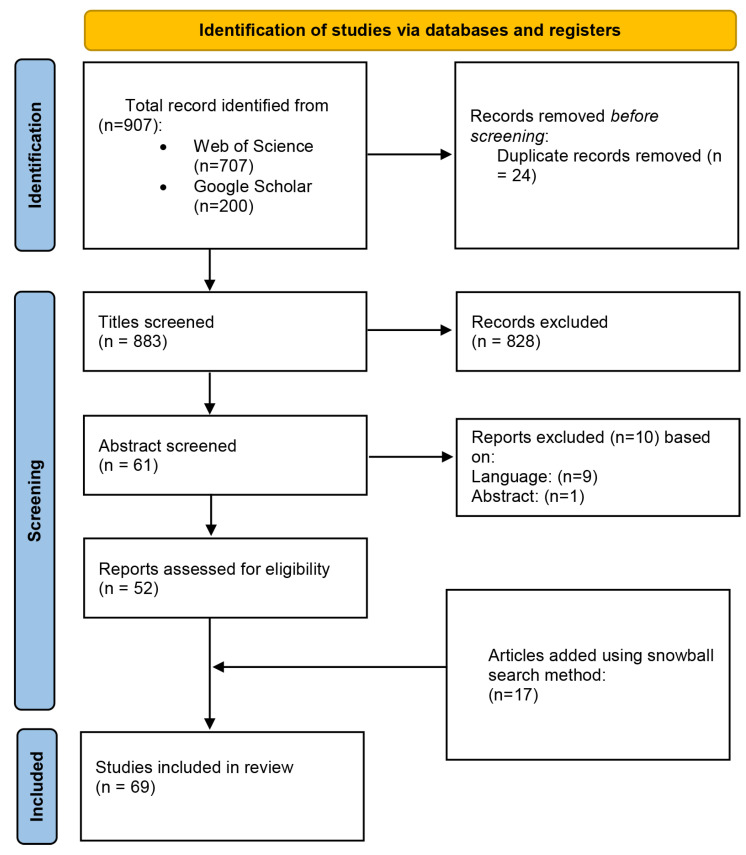
PRISMA Flow Diagram.

This systematic review was conducted in accordance with the recommendations of the Preferred Reporting Items for Systematic Reviews and Meta-Analyses (PRISMA) statement (Figure 1) [34].

Studies were required to be written in English, Portuguese, or Spainish. Participants had to rate images using a dimensional and/or categorical approach.

Firstly, WebOfScience was searched with the terms “International Affective Picture System” (all fields), and the articles that matched the inclusion criteria were selected. For the further research of grey literature, the first 200 entrances of Google Scholar with keywords “International Affective Picture System” AND “Validation” were analysed. Further articles were found by reviewing the bibliographic references of the articles selected (snowball search method [35]) (See Table 1).

IAPS validations were found for Germany [36,37], Taiwan [38], China [39,40], Russia [41,42], Republic of Korea [43], and France [44], but no english translation was available. Furthermore, a report for the Iranian population was found, but it was excluded due to being an abstract [45].

**Table 1:** Overall characteristics of studies included in Systematic Review. Abbreviations: Arousal (A), Bivariate Evaluation and Ambivalence Measures (BEAMs), Electroencephalography (EEG), Electromyography (EMG), Heart-Rate Variability (HRV), Modified Rating Scale (MRS), Not Applicable (N.A.), Not Reported (N.R.), Picture Presentation Type (PPT), Self-Assessment Manikin (SAM), Skin Conductance Level (SCL), Valence (V), Valence-Arousal (VA), Valence-Arousal-Dominance (VAD).

Ref.	Country	Year	N	Age Group	Age	Occupation	Stimuli	Sets	Ratings	Sensors	PPT	SAM	MRS	Written Language
[46]	Argentina	2016	125 (84 ♀ 41 ♂)	Adults	21.6 ± 5.13	Uni. Students	59	19	VAD	-	Group	Paper	-	Spanish
[47]	Argentina	2020	646 (342 ♀ 304 ♂)	Adults	25.86 ± 7.52	Uni. Students	412	3, 8, 9, 10, 11, 12, 15	VAD	-	Group	Paper	-	Spanish
[48]	Argentina	2015	524 (278 ♀ 246 ♂)	Adults	23.32 ± 6.69	Uni. Students	358	1, 2, 4, 5, 7, 14	VAD	-	Group	Paper	-	Spanish
[49]	Argentina	2022	141 (67 ♀ 74 ♂)	Children and Adolescents	11.16 ± 2.16	School Students	60	N.R.	VA	-	Group	Paper	-	Spanish
[50]	Argentina	2017	141 (67 ♀ 74 ♂)	Children and Adolescents	11.16 ± 2.16	School Students	60	N.R.	VA	-	Group	Paper	-	Spanish
[51]	Belgium	2001	80 (50 ♀ 30 ♂)	Adults	19.16 ± 1.87	Uni. Students	60	N.R.	VAD	-	Group	Paper	-	English
[52]	Bosnia	2013	72 (55 ♀ 22 ♂)	Adults	N.R.	Uni. Students	60	N.R.	VAD	-	Group	Paper	-	English
[53]	Brazil	2019	30 (13 ♀ 17 ♂)	Adults	44.6 ± N.R.	Medical Doctors	36	N.R.	VA	-	Individual	Paper	-	Portuguese
[54]	Brazil	2008	448 (269 ♀ 179 ♂)	Adults	24.2 ± 7.8	Uni. Students	240	13 - 16	VAD	-	Group	Paper	-	English
[55]	Brazil	2016	100 ♀	Adults	25.07 ± 7.175	Uni. Students	105 (80 IAPS)	N.R.	VA, Categorical	-	Group	Paper	-	English
[56]	Brazil	2008	48 (42 ♀ 6 ♂)	Elderly	68.65 ± 6.7	Third Age Open Uni. Students	71	N.R.	VA	-	N.R.	Paper	-	Portuguese
[57]	Brazil	2011	187 (111 ♀ 76 ♂)	Elderly	68.3 ± 6.99	N.R.	702	N.R.	VA	-	Group	Paper	-	Portuguese
[58]	Brazil	2008	448 (269 ♀ 179 ♂)	Adults	24.2 ± 7.8	Uni. Students	240	13–16	VAD	-	Group	Paper	-	Portuguese
[59]	Brazil	2018	161 (69 ♀ 92 ♂)	Adolescents	15 ± 2.2	School Students	182	N.R.	VA	-	Group	Paper	-	English
[60]	Brazil	2005	1062 (698 ♀ 364 ♂)	Adults	22.8 ± 4.6	Uni. Students	707	1-12	VAD	-	Group	Paper	-	English
[61]	Brazil	2007	24 (12 ♀ 12 ♂)	Adults	N.R.	Uni. Students	32	N.R.	VA	Facial EMG, SCL, HR, and peripheral temp.	Individual	N.R.	-	English
[62]	Chile	2010	135 (88 ♀ 47 ♂)	Adults	20.13 ± 2.29	Uni. Students	188	N.R.	VA	-	Group	Paper	-	English
[63]	Chile	2016	60 (30 ♀ 30 ♂)	Adults	22.3 ± 3.2	Uni. Students and 3 Finished High School	146	N.R.	VAD, Categorical	-	Individual	Paper	-	English
[64]	Chile	2011	208 (124 ♀ 84 ♂)	Adults	19 ± 1.2	Uni. Students	119	7;14	VAD	-	Group	Paper	-	English
[65]	China	2015	120 (53 ♀ 67 ♂)	Adults	21.35 ± 1.58	Uni. Students	816	N.R.	VA	-	Individual	Computer	-	English
[66]	China	2017	493 (274 ♀ 219 ♂)	Adults	19.66 ± 2.01	Uni. Students	108	N.R.	Emotion intension 9-point (0–8)	-	Individual	N.A.	-	English
[67]	China	2016	126 (86 ♀ 40 ♂)	Elderly	67.3 ± 4.96	N.R.	942	All sets (excluding erotic)	VA	-	Individual	Computer	-	English
[68]	Colombia	2019	1222 (699 ♀ 523 ♂)	Adults	20.39 ± 2.60	Uni. Students	240	15–18	VAD	-	Group	Paper	-	Spanish
[69]	Colombia	2019	447 (295 ♀ 149 ♂, 3 N.R.)	Adults	20.36 ± 2.74	Uni. Students	200	N.R.	Categorical	-	Individual	N.A.	-	English
[70]	Colombia	2011	404 (229 ♀ 175 ♂)	Adults	22.3 ± 5.2	Uni. Students	248	13, 14, 19, 20	VAD	-	Group	Paper	-	Spanish
[71]	Finland	2010	25 ♀	Adults	N.R.	Uni. Students	48	N.R.	VA	HRV, Eye Tracking, Facial Expressions	Individual	Oral Report	-	English
[72]	Finland	2013	14 ♂	Adults	N.R.	Uni. Students	48	N.R.	VA	HRV, Eye Tracking, Facial Expressions	Individual	Oral Report	-	English
[73]	Finland	2008	5 ♀	Adults	N.R.	Uni. Students	48	N.R.	VA	HRV, Eye Tracking, Facial Expressions	Individual	Oral Report	-	English
[74]	Finland	2010	25 ♀	Adults	N.R.	Uni. Students	48	N.R.	VA	HRV, Eye Tracking, Facial Expressions	Individual	Oral Report	-	English
[75]	Finland	2013	44 (25 ♀ 19 ♂)	Adults	N.R.	Uni. Students	48	N.R.	VA	HRV, Eye Tracking, Facial Expressions	Individual	Oral Report	-	English
[76]	Germany	2006	27 (Sex N.R.)	Adults and Elderly	49.3 ± 4.62	N.R.	702 (54 rated)	N.R.	VA	EEG	Individual	N.R.	-	English
[77]	Germany	2011	41 ♀	Adults	30.0 ± 7.6	Uni. Students and others (N.R.)	120 (20 IAPS)	N.R.	VAD	-	N.R.	N.R.	-	English
[78]	Germany	2009	156 (95 ♀ 61 ♂)	Adults and Elderly	41.9 ± N.R.	Uni. Students and others (N.R.)	172	N.R.	VA	-	Individual	Paper	-	English
[79]	Germany	2011	104 (53 ♀ 51 ♂)	Adults and Elderly	46 ± 3.9	Uni. Students and others (N.R.)	172	N.R.	VA, Categorical	-	Individual	Computer	-	English
[80]	Germany	2012	191 (95 ♀ 96 ♂)	Adults	23.6 ± 2.8	Uni. Students, Workers and others (N.R.)	298	N.R.	VA, Categorical	-	Individual	Computer	-	English
[81]	Germany	2008	106 (52 ♀ 54 ♂)	Adults and Elderly	47.42 ± 3.485	N.R.	504	N.R.	VA	-	Individual	Computer	-	English
[82]	Hungary	2010	187 (146 ♀ 41 ♂)	Adults	19.91 ± 1.34	Uni. Students	239	N.R.	VAD	-	Group	N.R.	-	English
[83]	India	2013	80 (36 ♀ 44 ♂)	Adults	23.7 ± 2.67	Uni. Students	100	N.R.	VAD	-	Individual	Computer	-	English
[84]	Israel	2011	38 (20 ♀ 18 ♂)	Adults	24.2 ± 2.9	Uni. Students	629	N.R.	VA	-	N.R.	N.R.	-	English
[85]	Japan	2019	62 (30 ♀ 32 ♂)	Adults and Elderly	44.72 ± 3.26	Uni. Students and others (N.R.)	120	N.R.	VA	-	Group	Paper	-	English
[86]	Lithuania	2015	103 (82 ♀ 21 ♂)	Adults	18–24 y	Uni. Students	60	20	VAD	-	N.R.	Paper	-	English
[87]	Malaysia	2017	72 (46 ♀ 18 ♂)	Adults	19.2 ± 1.68	N.R.	166 images (83 IAPS)	N.R.	VAD	-	Group	Paper	-	English
[88]	Mexico	2003	804 (Sex N.R.)	Adults	20.10 ± 3.69	Uni. Students	459 (266 IAPS)	N.R.	VAD + 2 subscale	-	Group	Paper	Valence rating inverted and Arousal was changed	Spanish
[89]	Mexico	2002	41 (21 ♀ 20 ♂)	Adults	24.8 ± 5.96	Uni. Students	700	N.R.	VA	-	Individual	Computer	5 point	Spanish
[90]	Mexico	2018	408 (220 ♀ 188 ♂)	Adults	19.81 ± 2.58	Uni. Students	238	13, 14, 19, 20	VAD	-	N.R.	N.R.	-	Spanish
[91]	Morocco	2020	100 (69 ♀ 41 ♂)	Adults	19.56 ± 1.21	Uni. Students	20	N.R.	V	-	N.R.	N.R.	-	English
[92]	Morocco	2018	120 (69 ♀ 51 ♂)	Adults	19.47 ± 0.67	Uni. Students	102	3;11	VAD	-	N.R.	Paper	-	English
[93]	Portugal	2015	2000 (1.419 ♀ 581 ♂)	Adults	21.57 ± 5.67	Uni. Students	1182	All	VAD	-	Individual	Paper	-	English
[94]	Serbia	2019	158 (73 ♀ 85 ♂)	Adults	19-21 y	Uni. Students	60	N.R.	VAD	-	Group	Paper	-	English
[95]	South Africa	2022	150 (75 ♀ 75 ♂)	Adults	21.6 ± 2.85	Uni. Students and others (N.R.)	340	N.R.	VA	-	Individual	Computer	-	English
[96]	Republic of Korea	2017	30 (15 ♀ 15 ♂)	Adults	23.8 ± 3.1	N.R.	15	N.R.	VAD	HRV	N.R.	N.R.	-	English
[97]	Republic of Korea	2009	104 (Sex N.R.)	Adults and Elderly	47.95 ± 3.65	N.R.	156	N.R.	V	-	Individual	N.R.	7 point	English
[98]	Spain	2001	715 (434 ♀ 281 ♂)	Adults	20.51 ± 3.40	Uni. Students	352	8-14	VAD	-	Group	Paper	-	Spanish
[99]	Spain	2008	45 (25 ♀ 20 ♂)	Adults	27.2 ± 9.5	Uni. Students	120	N.R.	VAD, Categorical	-	Group	Paper	-	English
[100]	Spain	1999	1102 (673 ♀ 429 ♂)	Adults	20.28 ± N.R.	Uni. Students	480	1-8	VAD	-	Group	Paper	-	Spanish
[101]	Spain	2013	811 (521 ♀ 290 ♂)	Adults	20.52 ± 3.73	Uni. Students	358	15–20	VAD	-	Group	Paper	-	Spanish
[102]	Spain/ Switzerland	2015	847 (541 ♀ 306 ♂)	Adults	22.91 ± 6.11	Uni. Students	60	N.R.	VA	-	Group	Paper	-	English
[103]	Turkey	2010	219 (59 ♀ 160 ♂)	Adults	21.17 ± N.R.	Elite Athletes	224	N.R.	VA	-	Individual	Computer	-	English
[104]	UK	2006	659 (340 ♀ 319 ♂)	Children	7–11 y	School Students	27	N.R.	VA	-	Group	Paper	-	English
[105]	US	2001	206 (106 ♀ 100 ♂)	Children, Adolescents and Adults	≥7 y	Uni. and School Students	60	N.R.	VAD	Facial EMG, HR, SCL	N.R. and Group	Paper & Computer	-	English
[106]	US	2005	66 (32 ♀ 34 ♂)	Adults and Elderly	18–71	Uni. Students and Retired	45	N.R.	VA	EEG, Facial EMG, HR	Individual	Computer	21-point	English
[107]	US	1995	60 (30 ♀ 30 ♂)	Adults	N.R.	Uni. Students	114	1–2	VAD, Categorical	Facial EMG	Group	Paper	-	English
[108]	US	2007	1302 (N.R.)	Adults	≥18 y	Uni. Students	703	N.R.	VA and Categorical/ Dimensional	-	Group	Paper	9-point	English
[109]	US	2014	13 (7 ♀ 6 ♂)	Adults	Median 34 y	N.R.	60	N.R.	VA	EEG (RREP)	Individual	Paper	-	English
[110]	US	2005	42 (23 ♀ 19 ♂)	Adults and Elderly	43.14 ± 3.96	Uni. Students	90	N.R.	VA	-	Individual	Computer	-	English
[111]	US	1998	509 (275 ♀ 234 ♂)	Adults	N.R.	Uni. Students	472	1–8	VAD and BEAM	-	N.R.	Paper	-	English
[112]	US	2005	140 (70 ♀ 70 ♂)	Adults	19.02 ± N.R.	Uni. Students	390	N.R.	Categorical	-	Individual	N.A.	N.A.	English
[113]	US	2000	46 (24 ♀ 22 ♂)	Adults and Elderly	47.4 ± N.R.	Uni. Students and others (N.R.)	27	N.R.	VA	Facial EMG	Individual	Computer	-	English
[114]	US	2004	34 (16 ♀ 18 ♂)	Adults and Elderly	50.91 ± 4.05	N.R.	64	N.R.	A (Not SAM)	fMRI	Individual	N.A.	Rating 1–4 in Arousal	English

## 3. Findings

Overall, characteristics of the 69 studies included in this review are reported in Table 1. Furthermore, in Table 2, a summary of the comparison between studies and the United States (US) normative study [22] is provided.

### 3.1. Localization

**Figure 2:**
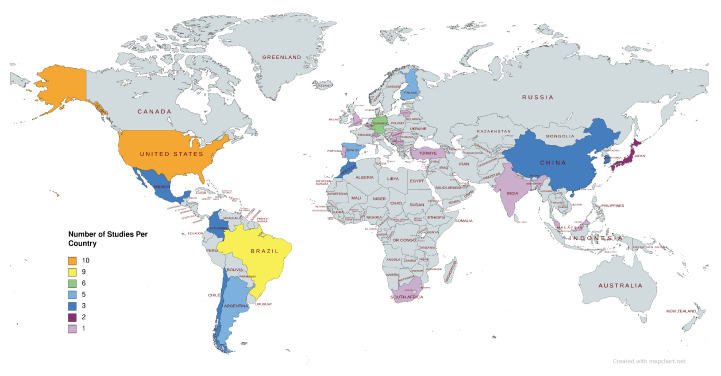
Topographic map of the world with studies represented.

The 69 studies selected for this review are spread across the world. Studies: (10) United States, (9) Brazil, (6) Germany, (5) Argentina, Finland, Spain, (3) Chile, China, Colombia, Mexico, Morocco, Republic of Korea, (2) Japan, (1) Belgium, Bosnia and Herzegovina, Hungary, India, Israel, Lithuania, Malaysia, Portugal, Republic of Serbia, South Africa, Switzerland, Turkey, and United Kingdom (Figure 2).

#### 3.1.1. Argentina

Irrazabal et al. [48] and Irrazabal and Tonini [47] provided normative data for the Argentine sample of the IAPS. Furthermore, Mina et al. [49] and Mina et al. [50] provided a normative rating for children and adolescents. The rating process was similar across studies with participants rating each picture in terms of valence, arousal, and dominance. Overall findings demonstrate that despite some differences with other cultures, IAPS is a reliable instrument for emotional elicitation in Argentina.

#### 3.1.2. Belgium

In a study by Verschure et al. [51], participants rated 60 IAPS pictures using SAM. The study found that the Flemish normative ratings were similar to the United States (US) ratings [22]. The affective ratings of the pictures in the Flemish sample correlated strongly with the US ratings for all SAM dimensions. Compared to the US sample, Flemish sample reported significantly lower levels of dominance. Furthermore, the distribution of the valence and arousal ratings demonstrated the expected boomerang shape as in US samples.

#### 3.1.3. Bosnia

In this study by Drace et al. [52], participants rated a sample of 60 IAPS pictures. The pictures were selected following the Verschure et al. [51] stratification procedure. A boomerang-shaped distribution was found, indicating the proper fit of IAPS for emotional elicitation in the Bosnian population. Results revealed a strong correlation between the affective ratings from the Bosnian sample, and the US ratings [22]. Bosnian sample rated significantly higher in arousal when compared with the US sample ratings.

#### 3.1.4. Brazil

In Brazil, multiple studies examined how different groups react emotionally to IAPS stimuli. The investigations assess the emotional reactions of various age groups and specific professions, such as medical professionals. These studies focused on the normative validation of the Brazilian population compared with the US sample. Overall results demonstrate that Brazilian sample ratings and US ratings are very similar in both groups.

In two studies [54,60], high correlations in valence, arousal, and dominance between the two samples were found. The Brazilian sample arousal rating was significantly higher than the US sample rating [22]. Lasaitis et al. [58] published an update to normative Brazilian norms, adding 240 more pictures. The analysis was focused on sex differences. Female ratings reported significantly less dominance than males. Another finding is that for unpleasant pictures, females reported lower valence and dominance and higher arousal. These results are consistent with the US sample. In a study [59] with adolescents, sex differences were found, when compared with males, females reported greater valence and lower arousal to pleasant pictures and lesser valence and more arousal to unpleasant pictures. Despite these differences, the study demonstrated similar results to previous validations.

In the elderly population [56,57], no statistical difference is found when compared with US data. Compared to Brazilian youths, most picture ratings become more extreme.

The elderly rated stimuli as more arousing compared to the younger Brazilian population. When compared with young US normative data, no statistical difference is found. Overall, these results demonstrate that arousal levels increased as pleasure decreased, resulting in a strong negative correlation.

Another study [55] investigated whether female nursing and social work students’ evaluations of surgical procedure pictures were influenced by their personal or professional relevance. Each participant rated the pictures dimensionally (valence and arousal) and discreetly (selecting a word to describe their feelings while viewing each stimulus). Results demonstrate a high correlation for both valence and arousal average scores compared to the US sample. Furthermore, the boomerang shape was found. From a dimensional point of view, no statistically significant differences were found between groups for IAPS pictures. The discrete evaluation demonstrated that social work students found the surgical procedure pictures to be more uncomfortable than the nursing students did. Additionally, the word “Neutral” was selected by 65.4% of Nursing students, while 54.2% of the Social Work group chose “Disgust”.

In another study [53] with medical-related participants, younger and older medical doctors’ ratings were compared. Results demonstrate that doctors who were older had more experience; they conducted consultations for longer periods of time and had more strong emotional responses to the stimuli. The emotional perception of the doctors and the general public was the same, though. The findings also indicated that compared to doctors who spend less time in the consulting room and divide their time among other tasks, doctors who work more hours per week in the consulting room had a less favorable perception of the stimuli.

Furthermore, a physiological validation was conduced, measuring facial electromyography activity, skin conductance, heart rate, and peripheral temperature [61]. Check Section 3.4.3 for physiological details.

#### 3.1.5. Chile

Three studies were found in Chile, and despite some differences, a boomerang distribution shape and strong correlations were found between the Chilean and US sample ratings, indicating a correct validation of IAPS in Chile.

In a study by Dufey et al. [62], participants rated 188 IAPS pictures using valence and arousal. Results demonstrated that compared with the US normative data, the Chilean sample reported lower levels of valence and a higher level of arousal. Strong correlations between the valence and arousal of Chile and US samples were found. Sex differences were found: males rated positive pictures as more arousing when compared to females’ ratings. In another study by Silva et al. [64], 208 participants rated 119 IAPS pictures (sets 7 and 14) using SAM. The authors compared the results to the Brazilian [54,60] and US [22] sample. When compared with Brazilian and US samples, Chileans rated pictures as significantly lower in arousal and higher in dominance. Sex differences were found within the Chilean sample, with females’ ratings being slightly higher in arousal and lower in valence.

Moreno et al. [63] identified fear-evoking pictures from the IAPS in a Chilean sample using categorical and dimensional evaluations. The study design follows a previous German study [80]. Results demonstrate that 30 of 64 pictures are identical between these studies. Furthermore, overall, the Chilean sample-rated pictures have a greater valence and arousal when compared to German ratings.

#### 3.1.6. China

Three studies were found in China. Overall, the Chinese sample rated high in arousal compared to the US sample [22]. However, cross-cultural compatibility seems feasible, as strong correlations between samples were found.

Huang et al. [65] compared the ratings of young adults from China and the US sample while viewing a standardized set of IAPS pictures. The main results focus on sex differences. Overall, the researchers found that females had more defensive ratings to aversive pictures, while males increased arousal ratings in erotic pictures. When compared to the US sample, Chinese participants rated lower in valence and higher in arousal, especially males [65]. In a novel study [66], researchers tried to access pure emotions in IAPS pictures. Participants rated 108 IAPS pictures with a set of emotions (disgust, erotism (or erotica), fear, happiness, sadness, and neutral emotions). Their task was to rate the intensity of perceived emotion using a 9-point (0–8) scale (neutral (0), weak (1), moderate (4) to strong (8)) for each picture presented. The authors used an exploratory and confirmatory factor analysis and found ten domains of emotion (mutilation–disgust, vomit–disgust, food–disgust, violence–fear, happiness, sadness, heterosexual couple–erotism, single male–erotism, single female–erotism, and neutral). A total of 59 pure emotion IAPS pictures were found. Sex differences were found: males rated high on couple–erotism and female erotism, while females rated higher on mutilation–disgust and sadness. [66] Another study by Gong et al. [67] reported a cross-age and cross-cultural analysis. Older adults rated 942 pictures using valence and arousal. The participant’s ratings were then compared with Chinese young adults [39]. Results demonstrated that although older persons find pleasant pictures to be the least appealing and negative pictures to be the most arousing, young adults find both negative and positive pictures to be more arousing than neutral pictures. The authors performed a cross-cultural comparison with the German population study [81]. German and Chinese older adults rated negative pictures as more arousing and positive ones as the least arousing. The ratings of valence and arousal of these groups were highly correlated, suggesting cross-cultural compatibility. Regardless, some differences were found, with older adult Chinese reporting significantly lower arousal for negative pictures and significantly higher arousal on positive pictures than older adult Germans ratings [67].

#### 3.1.7. Colombia

Three studies were found in Colombia with similar results to the US sample [22]. In these studies [68,70], participants had to rate IAPS pictures using SAM. Results demonstrated that the boomerang distribution shape was present in Colombian samples. Sex differences were found, with females scoring higher in arousal and more negatively for aversive stimuli, while males rated positive pictures as more positive and more arousing. Compared to US sample ratings, the results of Díaz et al. [70] demonstrate overall higher ratings of arousal and dominance while the results of Gantiva et al. [68] were similar to US samples.

In a study by De La Torre et al. [69], the authors further extended the Colombian IAPS validation in a discrete manner. A total of 200 pictures were rated with a 7-point emotion rating scale (1 = not at all; 7 = a lot) about how strongly the particular emotion was felt when viewing each picture (anger, disgust, fear, sadness, happiness, and satisfaction). The authors followed the analysis of Mikels et al. [112], enabling a direct comparison with the US sample. Results demonstrate more complex pictures (including more than one negative emotion) than Mikels and colleagues’, US validation [112]. The authors suggest that this difference is due to cultural differences that affect participants’ interpretation of IAPS pictures.

#### 3.1.8. Finland

Five studies were found in Finland [71,72,73,74,75]. These studies had the participants placed 65 cm from the monitor and verbally reported the valence and arousal of 48 pictures while HRV, facial expressions, eye tracking and voice were recorded. Furthermore, the data collected were shared between studies. Overall, pleasant stimuli seemed to produce a stronger emotional response compared to negative stimuli. Check Section 3.4.3 for physiological details.

#### 3.1.9. Germany

Six studies were found in Germany; results suggest that cultural and age differences may affect participants.

In three studies, young and older adults’ ratings were compared, reporting similar results and conclusions [78,79,81]. Results found that young adults revealed a stronger quadratic than linear relationship between valence and arousal. Compared with young adult ratings, elderly participants rated pleasant and neutral pictures as more pleasant and unpleasant pictures as more unpleasant. Furthermore, older adults rated pleasant, unpleasant, and neutral pictures as more arousing than young adults’ ratings. These results are in line with other findings in the literature [81,106,114]. The study by Grühn and Scheibe [81] compared the results with US ratings and found that in terms of valence, the ratings were closer to the neutral midpoint of the response scale: Positive pictures were rated as less positive compared to normative ratings, neutral pictures were rated as slightly more positive, and negative pictures were rated as similarly negative by older adults and less negative by young adults. By contrast, young and older adults arousal assessments were more intense (leaning toward the response scale’s endpoints) when compared to the normative evaluations: Negative pictures were assessed as more arousing than positive or neutral pictures.

In a study by Barke et al. [80], 298 IAPS pictures were rated using the SAM dimensions of valence, arousal, and a categorical rating (fear, anger, disgust, sadness, joy, love/erotic attraction, surprise, neutral, and extra input field ‘other’). Participants reported lower arousal when compared to the previous study with the German population [81] and the US validation [22]. Authors infer that maybe cultural differences could be the cause of this effect. Sex differences were also found; males rated pictures as more positive but less arousing than female ratings. Furthermore, females categorized pictures as fear-evoking more frequently than males, indicating that females have a greater propensity for evaluating situations as fear-evoking when compared to males.

A new set of erotic pictures was validated in a study by Jacob et al. [77]. The authors used 20 IAPS pictures with erotic content for comparison with the new stimuli. Female heterosexual participants rated each of the 120 pictures using SAM. Results demonstrated that the erotic picture sets’ valence was equal to that of the non-erotic positive IAPS pictures. Negative erotic pictures deviated significantly from the neutral category regarding arousal and dominance. These findings reveal that the new picture set is unsuitable for comparison to negative pictures. This concludes that for female heterosexual participants, the new pictures correspond directly to highly positive IAPS pictures but are higher in arousal and dominance.

Finally, a study by Wieser et al. [76] focuses on the relationship between neurophysiological markers and self-report ratings of young and elderly participants. Participants were exposed to an emotional rapid (3 Hz) serial visual presentation (RSVP) with 702 IAPS pictures in an alternating sequence concerning emotional arousal (i.e., high–low–high–low). After the RSVP, participants rated 54 IAPS pictures for valence and arousal. Some neurophysiological differences were found; however, no differences were found in the self-report for valence and arousal. Check Section 3.4.3 for physiological details.

#### 3.1.10. Hungary

In a validation study by Deák et al. [82], 239 IAPS pictures were rated using SAM. Results demonstrate that Hungarian females rated pictures with higher arousal and lower dominance compared to male ratings. The Hungarian mean ratings were strongly correlated with the US sample [22]. The Hungarian sample rated the pictures as being significantly higher in dominance.

#### 3.1.11. India

One study was found in India [83]. A total of 100 IAPS pictures were rated using SAM. For stimuli selection, the authors used the Verschuere et al. [51] stratification process. Results demonstrate a boomerang-shaped distribution between arousal and valence. Correlations between Indian and US ratings were positive and statistically significant for all dimensions. Mean differences were found when compared with the US sample [22]; Indian participants rated significantly higher in arousal and dominance. Overall, no significant sex differences were found. The authors caution researchers to take into account the arousal and dominance values when using IAPS to study the Indian population, as some cross-cultural variations exist.

#### 3.1.12. Israel

In a validation study by Okon-Singer et al. [84], 629 pictures were rated using valence and arousal. Results demonstrated a strong positive correlation between the mean valence and arousal ratings of North America and Israel. Compared to the US sample [22], Israeli students of both sexes rated pictures as less negative and less positive. Moreover, Israeli females gave the pictures higher arousal ratings than US females.

#### 3.1.13. Japan

A total of 31 older and 31 younger adults rated 120 pictures using valence and arousal [85]. Results demonstrate that no significant differences were found between older and younger samples for valence. Old adults rated pictures as more arousing than young ratings. In old adults, arousal ratings of negative pictures were higher than those of positive pictures. No significant difference was found between arousal ratings for neutral and positive pictures in adults. Positive correlations were found between young adults and old adults and the US sample [22]. In the three groups (younger adults, older adults, and US sample), valence and arousal were positively correlated.

#### 3.1.14. Lithuania

A total of 103 participants rated the 20th set (59 pictures) of IAPS using SAM [86]. A high correlation between Lithuanian and US samples [22] for all the SAM dimensions was found. The mean rating of arousal by the Lithuanian population was lower when compared with the US sample. Significant differences between sexes were found. Females rated pleasant pictures as more pleasant and unpleasant pictures as more unpleasant compared to male ratings. No differences were found for valence ratings in neutral pictures. Males scored a high arousal in both pleasant and neutral pictures than females. No sex differences were found in pictures or categories for dominance.

#### 3.1.15. Malaysia

In one study in Malaysia [87], 72 participants rated 166 pictures (83 were IAPS and the remaining were internet pictures) using valence and arousal [87]. Malaysian participants reported a significantly higher arousal level when compared to the US sample [22]. Strong correlations were found between Malaysian ratings and US ratings.

#### 3.1.16. Mexico

Three studies were found in Mexico, displaying an overall boomerang-shaped affective space and some variations in mean ratings across cultures. In a study by Castilho-Parra et al. [89], 700 pictures were rated using valence, arousal, and reaction time. Results demonstrate that for pictures with affective content, the reaction time is shorter. Compared to male ratings, female ratings were more extreme, leaning towards either positive or negative, and were rarely neutral. Overall mean ratings were similar to US ratings [22], with a few exceptions in some pictures.

In Chayo-Dichy et al. [88], 459 pictures were rated using a modified version of SAM. This modified version has two extra subscales: “Moral Content” (1—no moral content; 9—intense moral content) and “Evaluation Difficulty” (1—Very difficult to evaluate; 9—No difficulty evaluating the existence of moral content). Furthermore, in the valence dimension, instead of the traditional 1—Very Negative to 9—Very Positive, participants are presented with an inverted version, starting with 1—Very Positive to 9—Very Negative. Another difference is that the Arousal dimension ratings were changed: instead of the traditional 1—Very Calm to 9—Extreme Arousal, participants are presented with a modified version, in which 1—Extreme arousal, 9—Very Calm, and 5—Neutral.

In Romo-Gonzales and colleagues’ [90] study, 408 participants rated 238 IAPS pictures (sets 13, 14, 19, and 20) using SAM. The authors compared the results obtained with the US and Colombian [70] validations. Overall, the results demonstrate a boomerang shape in the affective space that aligns with previous validations. Sex differences were found. When compared to females, male dominance ratings were higher. Furthermore, all correlations were statistically significant between them except the nonstatistical significant relationship between arousal and dominance in females. Compared to the US and Colombian samples, the Mexican sample scored higher in valence, lower in arousal, and higher in dominance.

#### 3.1.17. Morocco

Both studies by Bandadi et al. [91,92] examine nursing students. The [92] study explores the effect of clinical traineeship on emotional dimensions. The pre and post-traineeship both consider negative pictures as unpleasant. A significant difference is found in valence, with the pre traineeship group rating lower in valence. A boomerang shape distribution was found. In the second study from 2020 [91], final-semester and first-semester nursery students rated negative pictures. Results demonstrate that final-semester students rated the negative pictures as less unpleasant compared to first-semester student ratings. Some pictures were rated significantly differently from the US sample ratings [22].

#### 3.1.18. Portugal

In a validation study by Soares et al. [93], 1,182 pictures were rated using the SAM. The normative values of the IAPS for Portugal are correctly distributed in the affective space of valence and arousal, according to the results, which also demonstrated the typical boomerang-shaped distribution observed in earlier studies. Significant sex differences were found. Males rated IAPS stimuli with higher levels of dominance and valence while females reported higher levels of arousal. In contrast to participants from the US [22], Spain [98,100], and Brazil [54,60], study participants from Portugal rated pictures from the IAPS with lower levels of valence. In contrast, they found that IAPS pictures had higher levels of arousal than those from the US [22] and Chile [62,64], but lower levels of arousal than those from Spain [98,100], Brazil [54,60], and India [83]. In the dominance dimension, Portuguese participants gave IAPS pictures lower ratings than US and Bosnia-Herzegovinian participants, but higher ratings than Spanish participants. In addition, males rated IAPS pictures with higher levels of valence and dominance than females, while females demonstrated higher levels of arousal than males, regardless of the IAPS standardization.

#### 3.1.19. Serbia

In a study by Grabovac and Deák [94], a sample of participants from Serbia and Hungarians living in Serbia rated 60 IAPS pictures using SAM. The stimuli were the same as those of the stratification process of Verschuere et al. [51]. The authors found that despite the highly correlated mean ratings with the US [22], Bosnia [52], and Hungary [82], the Serbian and Hungary from Serbia groups had the highest correlation. In comparison to the US group and the Hungarian group from Hungary, the Hungarian group from Serbia scored higher on arousal. Moreover, the Hungarian group from Serbia scored less favorably in terms of dominance than the Hungarian group from Hungary. This shows that the Hungarian group from Serbia is more sensitive to the emotional effects of their surroundings and has a lower threshold for arousal. Sex differences were also found, with females rating higher in arousal and lower in dominance when compared with males.

#### 3.1.20. South Africa

In a study by Nestadt et al. [95], a new standardized emotional elicitation dataset was introduced. The South African Affective Picture System (SA-APS) was developed for use in low-and middle-income countries by modifying the IAPS to include culturally relevant stimuli and more diverse groups of people. The study discovered that, especially in terms of valence, the ratings of the SA-APS pictures were more closely aligned with US normative standards [22] than those of the original IAPS pictures. The socioeconomic status (SES) of the participants and their ratings of the IAPS pictures varied, with lower SES participants giving the pictures lower ratings. Sex and SES were found to be significant predictors of the participants’ ratings through regression modeling, and regression-based norms were developed for both picture sets. Overall, the findings indicate that the SA-APS might be a better alternative to IAPS in South Africa because its ratings were comparable and closer to North America’s.

#### 3.1.21. Republic of Korea

A study by Kwon et al. [97] examined whether older Koreans display the positivity effect, a phenomenon where older adults in Western cultures have better memories for positive than negative material. The study involved showing pictures from the IAPS to younger and older Korean participants, testing their memory and recognition of the pictures, and asking them to rate the pictures for valence. To account for potential age and cultural differences in the interpretation of the stimuli, pictures were categorized based on valence ratings provided by younger and older Korean participants. Results demonstrate that the younger Korean group did not deviate significantly from the normative US sample [22], showing minimal cross-cultural interpretational variability. The valence ratings of older Koreans, however, were significantly different from those of younger Koreans and the IAPS, with older Koreans interpreting negative pictures less negatively and neutral or positive pictures more positively.

In a study involving physiological measurement, the IAPS was used to assess emotions using heart rate variability (HRV) [96]. Five pictures from the IAPS were chosen by the researchers for pleasant, unpleasant, and neutral categories. Participants rated each with SAM while HRV was collected. Results demonstrated that high arousal pictures should be used in experiments measuring emotion change using HRV. Check Section 3.4.3 for physiological details.

#### 3.1.22. Spain

Four studies were found in Spain, all showing close results to the US sample ratings [98,100,101]. In three of the studies, sets of IAPS pictures were rated using the SAM by university students. The first study used 480 pictures in sets 1 through 8, the second study 352 pictures in sets 9 through 14, and the third study 358 pictures in sets 10–15. Females consistently rated the pictures higher in arousal and lower in dominance than males in all three studies, with the Spanish sample rating the pictures higher in arousal and lower in dominance than the US sample. Between the three studies, as well as between the Spanish and US samples, the findings were remarkably consistent. A study by Javela et al. [99] found ratings similar to the Spanish normative and US normative ratings.

#### 3.1.23. Spain/Switzerland

IAPS was used with a sample of Spanish and Swiss participants to evaluate the link between anxiety, impulsivity, and emotions [102]. Females performed better in the valence/arousal picture groupings, particularly in the negative valence–high arousal category, according to the scientists, who separated the IAPS photos into five groups. In both countries, females scored significantly higher in impulsivity and anxiety.

#### 3.1.24. Turkey

One study was found in Turkey [103]. A total of 224 IAPS pictures were rated using SAM dimensions of valence and arousal. Significant correlations were found between Turkish and US [22] sample ratings for valence and arousal. Mean ratings were similar between Turkey and US samples.

#### 3.1.25. United Kingdom

In a validation study by Sharp et al. [104], children rated 27 IAPS pictures. Children rated pleasant pictures as more arousing than unpleasant pictures. Significant sex differences were found for valence ratings of unpleasant pictures, with girls rating higher than boys. Valence ratings were identical to the US sample ratings [22,105]. The authors reported that the arousal ratings for unpleasant high-arousal pictures were lower when compared to the US normative data.

#### 3.1.26. United States

Ten studies were found in the United States. These studies deeply explored the inter-age differences from a categorical and dimensional point of view, as well as the physiological elicitation capabilities of IAPS.

Multidimensional normative evaluations for the IAPS were presented by Libkuman et al. [108] in 2007, namely categorical (happiness, surprise, sadness, anger, disgust, and fear), dimensional and dimensions of consequentially, meaningfulness, familiarity, distinctiveness, and memorability. Results demonstrate similar valence and less arousing ratings compared to the US norms. Backs et al. [110] contrasted the self-assessment manikin assessments of emotive pictures in younger and older persons, finding that both younger and older adults differed from the norms for valence for pleasant pictures, but there were no significant differences between the two groups. Younger adults find pleasant-aroused pictures as more pleasant and arousing than older adults [110]. Using the IAPS, Ito et al. [111] investigated the trajectories of emotional reactions and their exploration in the affective space. Data on the emotional category for photographs from the IAPS were gathered by Mikels et al. in 2005 [112] to provide a descriptive categorization of IAPS based on the Ekman [3] emotional model.

The following studies used physiological measurements (for more information on these check Section 3.4.3): Children’s emotional responses to affective photographs were examined by McManis et al. [105], who reported that different types of pictures elicited diverse physiological, verbal, and behavioral responses [105]. According to Smith et al. [106], older adults reported greater overall pleasure and valence than young adults and the electroencephalography (EEG) data shows a decreased N1 and P3 event-related potentials (ERP) amplitude (N1 is a sensory processing-related negative-going deflection that usually appears 100 ms after a stimulus. P3 is a positive-going deflection that usually appears 300 ms after a stimulus and is linked to cognitive functions such as working memory, attention, and decision-making [115,116]), facial Electromyography(EMG) activity, and heart rate deceleration. Davis et al. [107] used the IAPS to conduct a dimensional, categorical, and electromyographic examination of the human effect brought on by static color slides, finding that reports are similar within and in different cohorts, which further validated the high replicability of IAPS. The age-invariance in the asymmetry of stimulus-evoked emotional facial muscle activity was examined by Reminger et al. [113]. Participants viewed and rated positive, neutral, and negative images while their facial activity was recorded, comparing young and old adults’ subjective ratings and facial EMG activity [113]. Results demonstrate that the age group had no discernible impact on either subjective evaluations or EMG patterns. Finally, Mather et al. [114] investigated the amygdala’s reactions to emotionally charged stimuli in both older and younger adults, finding that while this was not the case for younger adults, seeing positive pictures induced more amygdala activation than seeing negative pictures. In the study by Chenivesse et al. [109], the authors explored the effect of negative emotion on respiratory sensory gating. Respiratory-related evoked potential RREP was used as a measure (RREP are recorded using EEG and measure cortical activity in reaction to respiratory stimuli such as short inspiratory occlusion or breathing against inspiratory resistive loads. This offers details on the earliest phases of sensory afferent respiratory information processing in the cortex, including the original arrival and subsequent processing of this information [117,118]).

### 3.2. Participants’ Characterization

The 69 studies reviewed had a combined sample of 19,463 participants with a mean age of 28.67 years. Among the participants, 10,317 were female, 6913 were male, and the sex of 2237 participants was not reported. Studies with a total or partial sample of adults account for 88% of these, 20.28% have a partial or total sample of old adults, and 7.25% have a partial or total sample of children and adolescents. Studies’ age group: (51) Adults; (10) Adults and Elderly; (3) Elderly, (2) Children and Adolescents; (1) Adolescents; (1) Children; and (1) Children, Adolescents, and Adults. A total of 75.81% of the studies (53 out of 69) comprised a total or partial sample of university students, of which 45.3% (24 studies) had a partial or full sample of psychology students, and 5230 were female and 2701 were male. Furthermore, 30% (21) of the studies reviewed used some neuropsychological instrument for participants’ assessment (Table 3).

### 3.3. Studies Timeline

**Figure 3:**
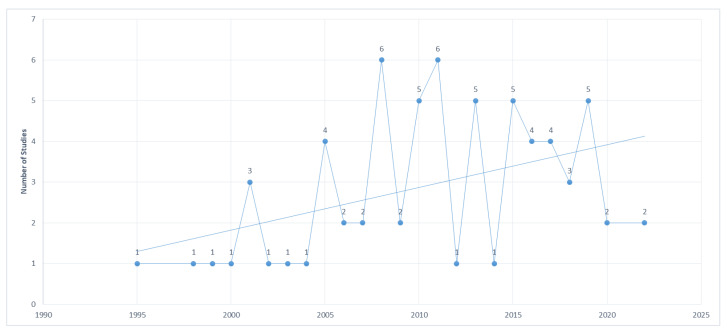
Number of studies across the years.

The year range of the 69 studies reviewed is 1995 to 2022. A clear trend is that the number of publications has increased over time. By dividing the data into five-year intervals, this becomes clear. Number of studies: 4 (1995–2000), 10 (2000–2005), 17 (2005–2010), 18 (2010–2015), 18 (2015–2020), and 2 (2020–2022). The highest number of studies was in 2008 and 2011, totaling 6. The most common number of studies per year is 1 with 9 data points (1995, 1998, 1999, 2000, 2002, 2003, 2004, 2012, and 2014) followed by 2 with 5 data points (2006, 2007, 2009, 2020, and 2022), 5 with 4 data points (2010, 2013, 2015, and 2019), 4 with 3 (2005, 2016, and 2017) and, lastly, 6 and 3 with 2 data points each (2008 and 2011; 2001 and 2018). Since its introduction in the 1990s, IAPS is still being validated. Since 2008, a crescent trend is found, as demonstrated in Figure 3.

### 3.4. Stimuli Characterization

**Figure 4:**
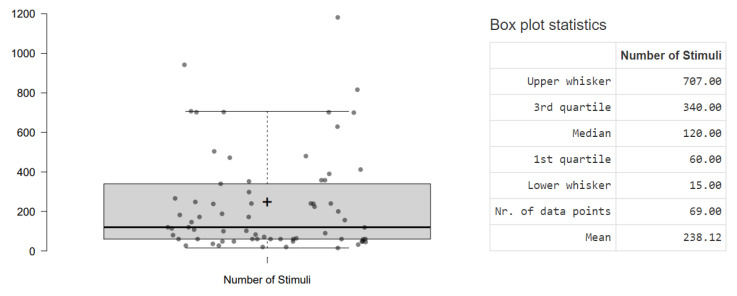
Boxplot of Number of Stimuli used; the dots represent each study.

The reviewed studies used a minimum of 15 IAPS stimuli [96] and a maximum of 1182 [93]. The most frequent number of stimuli used is 60, corresponding to 5.08% of the IAPS database, by 9 studies [49,50,51,52,86,94,102,105,109]. The data are displayed in Figure 4.

For stimuli selection, some articles used the method of stratification introduced by Verschuere et al. [51]. In this method, in order to select 60 pictures from a pool of 604 stimuli, a three-step stratification procedure was used. First, for each SAM dimension, three levels (low, average, and high) were created, resulting in 27 strata. Second, using the normative values, each picture was classified into one stratum. Lastly, the size of the strata was compared to the whole dataset for defining the percentage of pictures. Using the author’s example, 25 pictures out of 604 is 4%, then 4% of the stratum is selected for the final sample.

In this review, two articles utilized the stratification method for image selection [52,83], and one study utilized the same 60 images as the original stratification process [94]. However, the remaining articles resorted to full sets of selection or a combination of images from different sets, indicating a lack of consensus on a standardized image selection approach.

#### 3.4.1. Stimuli Presentation

For the classification of studies, two categories were created: (1) Group approach in which a group of participants viewed the pictures on the same screen, usually a projection. (2) Individual approach, with each participant having their own screen, usually a monitor. Overall, 30 studies (43.48%) used a group approach; in one of these studies, participants viewed the pictures on a stand and show [104]. A total of 29 studies (42.03%) used the individual approach. In one study, participants viewed the images in a printed fashion [78]. Nine studies (13.04%) did not report the display information. Finally, one study was composed of two experiments. In experiment 1, no display info was reported, and in experiment 2, a group approach was used.

#### 3.4.2. Rating

In this review, 65 (94.20%) studies used SAM for the emotional rating. The most common usage of SAM is in a pen-paper fashion. Although SAM is composed of three dimensions, valence (V), arousal (A), and dominance (D), its utilization is often partial, most of the time by removing dominance. A total of 11 studies (15.94%) collected categorical data using a categorical approach for picture rating based on Ekman’s emotion model (anger, disgust, fear, happy, sad, and surprise) [150]. Participants selected the emotion that best describes what they felt during the stimuli visualization or used an intensity scale combining emotion selection with a Likert-type scale for rating emotion intensity.

#### 3.4.3. Physiology

In this review, 14 studies (20.29%) included physiological measurements using multiple sensors. The most common physiological measurement was heart rate variability (HRV) in 6 studies [71,72,73,74,75,96]; eye tracking and facial expressions in 5 studies [71,72,73,74,75]; facial electromyography in 5 studies [61,105,106,107,113]; electroencephalography (EEG) and heart-rate in 3 studies [76,106,109]; skin conductance level (SCL) in 2 studies [61,105]; and finally respiratory-related evoked potentials (RREP) [109], functional magnetic resonance image (fMRI) [114], and peripheral temperature [61] in 1 study.

The following overall results describe the physiological reactions connected to various emotional stimuli. The findings from HRV and EEG indicate some age-related deficits in emotion discrimination. SCL and facial EMG studies imply that females may be more sensitive to unpleasant stimuli than males. According to the fMRI study presented [114], older and younger people react differently to positive and negative stimuli. Finally, studies on the heart rate indicate that seeing unpleasant images causes the heart rate to slow down. The physiological reaction to relaxing and pleasant images differs from that of neutral ones, according to changes in the peripheral temperature.

Heart Rate Variability: In a study conducted by Rantanen et al. [71,72], it was found that positive stimuli elicit a stronger emotional reaction than negative stimuli and that females, but not males, demonstrated significant differences in HRV responses to pleasant and unpleasant stimuli during the viewing phase. Another study [96] suggested that images with high arousal values were more strongly linked to changes in HRV. This study advises authors to use high-arousal images in experiments using HRV to assess emotional changes, as they elicit more clear physiological responses.

Viewing Distance and Facial Expressions: In the studies by Laukka et al. [74,75], eye tracking was used to access view distance. Men viewed pictures at the shortest distance and then slightly drew closer, but still, in comparison, viewed the pictures at the shortest distance. Women initially viewed pictures at the greatest distance and then slightly retreated, but the viewing distance remained the greatest. Men viewed pictures at an average distance when compared to neutral and pleasant pictures, while women maintained the greatest viewing distance when reporting unpleasant pictures. The authors also analyzed the videos recorded of the picture ratings for prediction of classification. A spatiotemporal local binary pattern descriptor was used. After feature extraction, a support vector machine with 10-fold cross-validation was used for classification. Two forms of ground truth were used: (a) participants’ rating and (b) form pictures. Results demonstrate that it is difficult to associate the participants’ assessment with facial expressions resulting from poor classification [73].

Facial EMG: McManis et al. [105] found that children displayed more corrugator EMG activity when viewing unpleasant pictures than pleasant ones. Girls responded more strongly to unpleasant images, displaying greater corrugator EMG activity differentiation and faster blink responses. When viewing unpleasant images, males tended to exhibit smaller changes in corrugator EMG activity and smaller blink reflexes. In the study by Smith et al. [106], corrugator EMG activity was lower in older people than in younger adults. Reminger et al., in 2000, found that the age group had no discernible impact on either subjective evaluations or EMG patterns and that positive stimuli increased the activity of the zygomatic muscles while negative stimuli increased the activity of the corrugator muscles [113]. Davis et al. [107] found that the EMG activity associated with affective responses was correlated with valence. However, the magnitude was lower than spontaneous facial movements using the same muscles. The IAPS slides were only moderately effective compared to the complete range of normal affective reactions [107]. Finally, the results in the study by Ribeiro et al. [61] demonstrate that the zygomaticus activity was lower during the viewing of low-arousal pleasant pictures than during the viewing of high-arousal pleasant pictures. Unpleasant stimuli led to a more strong zygomatical activity.

Electroencephalogram: The results found by Wieser et al. [76] suggest some signs of age-related deficits, since early emotional discrimination started to develop in 180 ms after the picture onset in young participants compared to 220 ms after the picture onset for elders. These findings indicate a possible delay of the affective discrimination with aging. Chenivesse et al. [109] found a decrease in the N1 peak amplitude associated with an attentional defect and a reduction in the gating of the respiratory-related evoked potentials (RREP). The N1 peak was the two effects that watching unpleasant images had on the RREP. The latter result leads to the hypothesis that the over-perception of respiratory sensations experienced by some patients may be linked in part to a decrease in the respiratory sensory gating caused by emotions. Smith et al. [106] found that N1 and P3 amplitudes of the startle-elicited Event-Related Potentials (ERP) were lower in older adults. Compared to younger adults, older adults also blinked more frequently in reaction to unpleasant images, but this age effect was not observed for neutral or pleasant images.

Heart Rate: Ribeiro et al. [61] found that compared to neutral and pleasant stimuli, there was a secondary slowing of the heart rate after watching unpleasant images. There was an early decceleration prior to this, followed by an acceleration. This difference was observed for pleasant images that were both relaxing and high-arousal, and, to a lesser degree, for neutral images. This indicates that this physiological response is connected to the pleasure dimension rather than the arousal dimension. Further research suggests that participants’ age influences the heart rate response to emotional pictures. Smith and colleagues [106] found that the heart rate decceleration was lower in older than younger adults. Lastly, McManis et al. [105] found that children’s heart rate changes differed depending on the images they were viewing, with a higher decceleration when viewing unpleasant images as opposed to pleasant ones. Overall though, this effect was insignificant. There were no sex disparities. Adults’ heart rate changes were unaffected by picture content.

Skin Conductance Level: McManis and colleagues [105] found that girls displayed greater changes in skin conductance levels than boys. Girls experienced a higher skin conductance magnitude than boys when viewing unpleasant images, but this difference was inconsequential for pleasant images. Adults’ skin conductance varied depending on the image content, varying more when viewing unpleasant images than when viewing pleasant or neutral images. Adults demonstrated no differences based on gender. Ribeiro et al. [61] found that even though the responses were comparable to those from pleasant high-arousal stimuli, unpleasant pictures caused higher skin conductance levels than pleasant, relaxing ones. According to the Brazilian population’s subjective assessments of arousal, some positive stimuli may lessen arousal even though they evoke the same reactions as unpleasant images. Furthermore, the correlation between skin conductance level and arousal is very strong [61].

Functional Magnetic Resonance Imaging: In terms of structural brain activity, Mather et al. [114] investigated the amygdala’s reactions to emotionally charged stimuli in both older and younger persons, finding that while this was not the case for younger adults, observing positive images induced more amygdala activation than seeing negative images [114].

Peripheral Temperature: The substantial difference in temperature change between relaxing and pleasant images and neutral ones suggests an increase in peripheral temperature. It appears that changes in pleasure, in general, impact this physiological variable because there was a tendency for comparable temperature changes for both pleasant high-arousal photographs and unpleasant ones compared to neutral ones. Due to the measure’s high response variability or low sensitivity, this impact may not have been significant [61].

## 4. Discussion

This review summarized 69 studies spread across almost two decades of research. Sample questions, stimuli selection, and physiology measurements are discussed. A comparison of the studies in other countries with a US sample is provided.

Despite the popularity and cross-cultural characteristics of IAPS, as pointed out by Nestadt et al. [95], most of the validations and studies using IAPS occur in developed countries. According to a 2008 report by the Population Reference Bureau (PRB), 1.2 billion people were living in developed countries and 5.4 in developing countries [151]. By this standard, the IAPS is not validated for most of the world’s population. Furthermore, the sample of the studies selected for this comprises university students, with the majority being psychology students. This fact might explain the sex imbalance found since, as reported by the American Psychological Association (APA), most psychology graduate programs have more female than male students [152]. The fact that most participants’ occupations are university students may also explain the imbalance in age groups. This bias is relevant and should be addressed in future research since emotional processing changes across our lifespan. The findings of this review demonstrate that, overall, most older adults tended to rate pictures as more arousing when compared to young ratings [56,57,67,76,78,79,81,85,97,106,110,113,114]. These findings are in line with previous reports in the literature under the “positivity effect”. This effect suggests that individuals tend to focus more on positive information and emotions with age and may even process negative information more positively. One possible explanation lies in alteration in the brain regions involved in emotional processing, such as the amygdala and prefrontal cortex [153,154,155]. According to Wieser et al. [76], when viewing positive pictures, older people had an increase in amygdala activation.

The physiological findings of the studies reviewed suggest that a clear activation of the fight or flight mechanism [156], resulting in a decreased heart rate and increased SCL, temperature change, startle response and change in view distance when faced with high-arousing stimuli [61,71,72,105] further solidifies the ability of IAPS for emotion elicitation in laboratory settings.

Most of the studies presented made a partial validation of IAPS. Maybe the reasons are due to the time- and resource-consuming task of running validation studies or the effectiveness selection processes such as the one introduced by Verschuere et al. [51] for selecting pictures representative of emotional space.

The fact that IAPS was introduced in 1995 and still new validations in different countries emerged indicates its impact and relevance for studying emotion. Most of the studies in this review compared the findings with the US normative data [22]. Overall, strong correlations were found between these samples. Nevertheless, some significant differences in mean ratings of valence, arousal, and dominance were found, indicating the presence of possible cultural differences [85,96]. Despite its widespread adoption in the field of emotion research, IAPS is not without its limitations. One notable limitation is that the resolution of the images is considered suboptimal by current standards. Additionally, some images may contain elements that are not recognizable to younger individuals, such as VHS tapes. Moreover, as noted in the Open Affective Standardized Image Set (OASIS) study by Kurdi et al. [7], using IAPS images in online studies is constrained by copyright concerns. Nevertheless, IAPS offers the advantage of being well-established in emotional elicitation research and could serve as a baseline for validating new images without copyright constraints. Finally, the static nature of the IAPS stimuli limits the extent to which they can elicit physiological responses compared to video stimuli, as reported in the study by Horvat et al. [157] comparing image and video elicitation.

Future research should concentrate on more diverse populations outside of academia. To provide a more precise understanding of emotional processing, researchers should also consider the integration, when possible, of physiological measurement. In future validation studies, it would be advantageous to ensure that the report on the validation data is readily accessible and user-friendly, for instance, through the use of a spreadsheet. This is especially critical, since some previous studies have presented the data solely within the text as a table or image format, which may not be as convenient for a further analysis.

Additionally, it is recommended that future reviews incorporate more studies utilizing physiological measurements, as such studies exist but were not included due to predetermined selection criteria. Overall, this systematic review provides important insights into the biases and limitations of the current research with IAPS. By addressing these limitations and incorporating more diverse and comprehensive measures, researchers can improve the generalizability of their findings, leading to a better understanding of emotional processing across different populations and cultures.

## Figures and Tables

**Table 2:** Summary of general dimensional ratings across different countries compared to US sample [22]. Abbreviations: No difference (N.D.).

Country	Reference	Valence	Arousal	Dominance
Belgium	[51]	N.D.	N.D.	Lower
Bosnia	[52]	N.D.	Higher	N.D.
Brazil	[54,60]	N.D.	Higher	N.D.
Brazil	[56,57]	N.D.	N.D.	-
Chile	[62]	Lower	Higher	-
Chile	[64]	N.D.	Lower	Higher
China	[65]	Lower	Higher	-
Colombia	[68]	N.D.	N.D.	N.D.
Colombia	[70]	N.D.	Higher	Higher
Germany	[80]	N.D.	Lower	-
Hungary	[82]	N.D.	N.D.	Higher
India	[83]	N.D.	Higher	Higher
Israel	[84]	Lower	N.D.	-
Lithuania	[86]	N.D.	Lower	N.D.
Malaysia	[87]	N.D.	Higher	N.D.
Mexico	[89]	N.D.	N.D.	-
Mexico	[90]	Higher	Lower	Higher
Morocco	[91,92]	N.D.	N.D.	N.D.
Portugal	[93]	Lower	Higher	Lower
Serbia	[94]	N.D.	Higher	N.D.
Republic of Korea	[97]	N.D.	N.D.	N.D.
Spain	[98,100,101]	N.D.	Higher	Lower
Spain	[99]	N.D.	N.D.	N.D.
Turkey	[103]	N.D.	N.D.	-
United States	[108]	N.D.	Lower	-

**Table 3:** Neuropsychological instruments used for participant evaluation in the reviewed articles. Abbreviations: 20-item Center for Epidemiologic Studies—Depression Scale (CES–D) [119], Alcohol Use Disorders Identification Test Consumption (AUDIT-C) [120], Animal Naming Task (ANT) [121], Antisocial Process Screening Device (APSD) [122], Beck Depression Inventory (BDI) [123], Clock Drawing Test (CDT) [124], Diagnostic and Statistical Manual of Mental Disorders (DSM) [125], Drug Abuse Screening Test (DAST-10) [126], Experienced Attention Deficits Self-Rating Inventory (FEDA) [127], Five Factor Personality Inventory (FFPI) [128], Future Time Limit (FTP) [129], Geriatric Depression Scale (GDS) [130], Hachinski Ischemic Score (HIS) [131], Impulsiveness, Venturesomeness and Empathy (IVE) [132], Instrumental Activities of Daily Living (IADL) [133], Korean Wahler Physical Symptoms Inventory (K-WPSI) [134], Korean Wechsler Adult Intelligence Scale (K-WAIS) [135], Mini-Mental State Examination (MMSE) [136], Patient Health Questionnaire for Depression-9 (PHQ-9) [137], Primary Care Post-Traumatic Stress Disorder Screen (PC-PTSD) [138], Positive and Negative Affect Schedule (PANAS) [139], expanded version of the Positive and Negative Affect Schedule (PANAS-X) [140], Self Depression Scale (SDS) [141], Sensitivity to Punishment and Sensitivity to Reward Questionnaire (SPSRQ) [142], Short Portable Mental Status Questionnaire (SHPMSQ) [143], State Trait Anxiety Inventory (STAI) [144], Strengths and Difficulties Questionnaire (SDQ) [145], Waterloo Handedness Questionnaire (WHD) [146], Wechsler Adult Intelligence Scale (WAIS) [147], Wortschatztest [Vocabulary test] (WST) [148], and Zuckerman–Kuhlman Personality Questionnaire (ZKPQ) [149].

Country	Reference	Instruments
Brazil	[56]	IADL, HIS, CDT
Brazil	[57]	IADL, HIS
China	[67]	CDT, GDS
Finland	[71]	STAI, TAS-20
Finland	[72]	STAI, TAS-20
Finland	[73]	STAI, TAS-20
Finland	[74]	STAI, TAS-20
Finland	[75]	STAI, TAS-20
Germany	[76]	SDS, MMSE, FEDA, PANAS
Germany	[78]	MMSE, STAI, WAIS
Germany	[79]	PANAS-X
Germany	[81]	WAIS, WST, PANAS
South Africa	[95]	PHQ-9, PC-PTSD, AUDIT-C, DAST-10
Republic of Korea	[97]	K-WAIS, K-WPSI, CES–D, PANAS, FTP
Spain	[102]	ZKPQ, IVE, SPSRQ
Turkey	[103]	FFPI
UK	[104]	SDQ, APSD, Questionnaires based on DSM
US	[106]	BDI, MMSE
US	[109]	STAI
US	[113]	WHD, MMSE, BDI
US	[114]	SHPMSQ, WAIS, ANT

## Data Availability

Not applicable.

## Abbreviations

The following abbreviations are used in this manuscript:

A	Arousal
ANT	Animal Naming Task
APSD	Antisocial Process Screening Device
AUDIT-C	Alcohol Use Disorders Identification Test Consumption
BDI	Beck Depression Inventory
BEAMs	Bivariate Evaluation and Ambivalence Measures
CDT	Clock Drawing Test
CES–D	20-item Center for Epidemiologic Studies—Depression Scale
CV	Computer Vision
DAST-10	Drug Abuse Screening Test
DSM	Diagnostic and Statistical Manual of Mental Disorders
EBIR	Emotional based image retrieval
EEG	Electroencephalography
EMG	Electromyography
ERP	Event-Related Potentials
FEDA	Experienced Attention Deficits Self-Rating Inventory
FFPI	Five Factor Personality Inventory
fMRI	Functional Magnetic Resonance Imaging
FTP	Future Time Limit
GDS	Geriatric Depression Scale
HIS	Hachinski Ischemic Score
HRV	Heart Rate Variability
IADL	Instrumental Activities of Daily Living
IAPS	International Afective Picture System
IVE	Impulsiveness, Venturesomeness and Empathy
K-WAIS	Korean Wechsler Adult Intelligence Scale
K-WPSI	Korean Wahler Physical Symptoms Inventory
MMSE	Mini-Mental State Examination
MRS	Modified Rating Scale
N.A.	Not Applicable
N.D.	No Difference
N.R	Not Reported
PANAS	Positive and Negative Affect Schedule
PANAS-X	Expanded version of the Positive and Negative Affect Schedule
PBR	Population Reference Bureau
PC-PTSD	Primary Care Post-Traumatic Stress Disorder Screen
PHQ-9	Patient Health Questionnaire for Depression-9
PPT	Picture Presentation Type
RREP	Respiratory Related Evoked Potential
SAM	Self Assessment Manikin
SCL	Skin Conductance Level
SDQ	Strengths and Difficulties Questionnaire
SDS	Self Depression Scale
SEEDs	Standardized Emotion Elicitation Databases
SHPMSQ	Short Portable Mental Status Questionnaire
SPSRQ	Sensitivity to Punishment and Sensitivity to Reward Questionnaire
STAI	State Trait Anxiety Inventory
US	United States
V	Valence
VA	Valence–Arousal
VAD	Valence–Arousal–Dominance
WAIS	Wechsler Adult Intelligence Scale
WHD	Waterloo Handedness Questionnaire
WST	Wortschatztest [Vocabulary test]
ZKPQ	Zuckerman–Kuhlman Personality Questionnaire
